# freeQuant: A Mass Spectrometry Label-Free Quantification Software Tool for Complex Proteome Analysis

**DOI:** 10.1155/2015/137076

**Published:** 2015-11-08

**Authors:** Ning Deng, Zhenye Li, Chao Pan, Huilong Duan

**Affiliations:** ^1^Department of Biomedical Engineering, Key Laboratory for Biomedical Engineering of Ministry of Education, Zhejiang University, Hangzhou 310027, China; ^2^General Hospital of Ningxia Medical University, Yinchuan 750004, China

## Abstract

Study of complex proteome brings forward higher request for the quantification method using mass spectrometry technology. In this paper, we present a mass spectrometry label-free quantification tool for complex proteomes, called freeQuant, which integrated quantification with functional analysis effectively. freeQuant consists of two well-integrated modules: label-free quantification and functional analysis with biomedical knowledge. freeQuant supports label-free quantitative analysis which makes full use of tandem mass spectrometry (MS/MS) spectral count, protein sequence length, shared peptides, and ion intensity. It adopts spectral count for quantitative analysis and builds a new method for shared peptides to accurately evaluate abundance of isoforms. For proteins with low abundance, MS/MS total ion count coupled with spectral count is included to ensure accurate protein quantification. Furthermore, freeQuant supports the large-scale functional annotations for complex proteomes. Mitochondrial proteomes from the mouse heart, the mouse liver, and the human heart were used to evaluate the usability and performance of freeQuant. The evaluation showed that the quantitative algorithms implemented in freeQuant can improve accuracy of quantification with better dynamic range.

## 1. Introduction

Study of complex proteomes has been widely applied in biomarker discovery, signaling pathway, and drug design [1–3]. In the cell, the development of disease is expressed by the changes of protein abundance; thus quantitative analysis for complex proteomes has increasingly become a critical way to investigate the mechanism of disease. Since the complex proteomes consist of a number of proteins with diversified functions, they pose a challenge for quantitative proteomics analysis. Over the past decade, mass spectrometry (MS) has been recognized as one of the most important techniques for proteomics science [4]. Specially, label-free quantitative approach based on spectral count has been widely used because of its ability to quantify large-scale proteomes. One of the representative methods of label-free quantification is Normalized Spectral Abundance Factor (NSAF) which was firstly proposed by Florens et al. [5]. This method uses protein length to normalize spectral count (SC) for improving the accuracy [6]. However, the NSAF method did not consider the shared peptides generated during the MS experiment [7]. Besides, the use of only spectral counts is not able to differentiate the MS spectra with different ion intensity, which leads to the systematic errors of quantification, especially for the low-abundant peptides. In this sense, selecting appropriate and valid MS spectral features is the key issue for MS-based quantification of complex proteome. On the other hand, an increasingly request of studying the complex proteome is to combine quantification and functional analysis together to reveal the unexplored mechanism in the cells. To propel this brand new analytic mode in proteomics science, innovative design of software tool is desiderated.

Nowadays, plenty of tools have paved the road to a new era in quantitative proteomics. John Yates' group [8] build a quantitative analysis tool for mass spectrometry-based proteomics called Census [9]. Census is compatible with many labeling strategies as well as with label-free analysis, single-stage mass spectrometry and tandem mass spectrometry (MS/MS) scans, and high- and low-resolution mass spectrometry data. Census supports multiple input formats and is extensively applied in quantitative proteomics. Besides, the APEX Tool described by Lu et al. [10] uses a modified spectral counting method which utilizes machine learning technique to arrive at protein abundance values with improved accuracy over traditional spectral counting techniques [11]. MaxQuant supporting labeling technique as well as label-free quantification is a quantitative proteomics software package designed for analyzing large mass-spectrometric data [12]. It uses correlation analysis and graph theory detects peaks, isotope clusters, and stable amino acid isotope-labeled peptide pairs as three-dimensional objects in *m*/*z*, elution time, and signal intensity space to quantify for proteomics. In conclusion, the abovementioned tools have gained recognition due to their distinctive accuracy and utility. However, there are no quantitative tools which combine quantification and functional analysis for complex proteomes and support label-free algorithms compatible with multiple MS spectral features. Therefore, it is of great significance to build a mass spectrometry label-free quantification software tool for complex proteomes analysis.

To systematically overcome this challenge, we developed a label-free mass spectrometry-based software tool for complex proteomes analysis, freeQuant, which combines quantification and functional analysis with biomedical knowledge extracting from the online protein functional analysis platform, g:Profiler [13]. freeQuant supports label-free quantitative analysis with multiple MS spectral features. The key part of freeQuant is the quantitative algorithms making full use of MS/MS spectral count, shared peptides, and ion intensity. freeQuant adopts normalized NSAF-based spectral count for quantitative analysis [6] and builds a new method for shared peptides to accurately evaluate abundance of isoforms. In addition, for proteins with low abundance, MS/MS total ion count (TIC) coupled with spectral count is included to ensure accurate calculation for protein abundance fold change. Another critical part of freeQuant is the combination of quantification and functional analysis which aims of comprehending and explaining functions and relationships of proteins in a large-scale manner. More importantly, the ease of use and accurate quantification as expected are also distinguished properties of freeQuant. We further performed the software evaluation using the MS spectral data of mitochondrial proteomes from our previous studies [14, 15]. We demonstrated that freeQuant with MS/MS TIC algorithm could alleviate the deficiency arising from SC and accurately evaluate protein abundance fold change. The strategy with shared peptides can improve the accuracy for low-abundant protein isoforms. In addition, freeQuant provides quantification analysis coupled with functional analysis, which is meaningful to deeply explore the functions and interactions of complex proteomes.

## 2. System Description

### 2.1. freeQuant: A Locally Installed Toolkit

**Figure 1:**
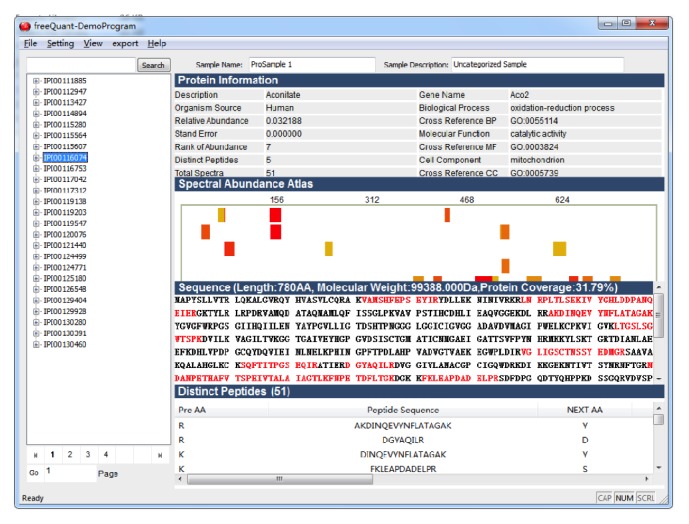
The Interface of freeQuant. Four modules were involved in freeQuant. First, protein information contains basic information of the current protein, such as protein name, relative abundance, and functional annotations. Second, spectral abundance atlas shows the distribution and relative abundance of identified peptides. Third, sequence shows the protein length, molecular weight, and protein coverage. Fourth, distinct peptide shows the correlative information of identified peptides.

freeQuant is a free and locally available toolkit dedicated to the quantitative and functional analysis of complex proteomes, and the main interface of freeQuant is shown as Figure 1. freeQuant was developed by C++ language using Microsoft Visual Studio 2010. It runs in Microsoft Windows 7 (2 GB memory or higher, 128 GB HDD or higher) and can be freely downloaded from ftp://ftp.vico-lab.com/. The locally installed version, which provides a simple and user-friendly interface, runs independently on the user's PC and it is more powerful to process large-scale data. freeQuant consists of four parts: data loading, quantification, functional analysis, and results displaying, as supplementary Figure S1 in Supplementary Material available online at http://dx.doi.org/10.1155/2015/137076 shows. The inputs of freeQuant are the FASTA database, MS data, and MS/MS files. Importantly, the kernel of freeQuant consists of two well-integrated modules: (1) label-free quantitative analysis using the MS spectral features; (2) mining biomedical knowledge for functional annotation. These two modules are highly interactive and cross-linked to allow fruitful analysis of complex proteomes.

### 2.2. Label-Free Quantitative Algorithm

To ensure the quantitative accuracy of complex proteomes with the freeQuant toolkit, we utilized two MS spectral features, the SC and the TIC, to quantify identified proteins in a large-scale manner. Firstly, the normalized spectral abundance factor (NSAF) based spectral count was adopted for quantification of complex proteomics, as (1) shows. All spectral counts are summed for each identified protein and then divided by protein length, generating the values of spectral abundance factor (SAF); the SAF value of each identified subunit is then normalized against the sum of all SAFs within an individual biological sample, resulting in the normalized SAF (NSAF) value; all NSAF values are then calculated separately for all biological samples. The average value of NSAF for each identified protein is employed for further quantitative and biological analysis. Secondly, a new method with shared peptides is proposed to explore how to accurately estimate abundance of isoforms. We used distinct peptides as proportional factor and allocate shared peptides to protein isoforms. Corresponding with NSAF, protein length was employed to rectify distinct peptides and obtain proportional factor by normalizing distinct peptides similarly, and then shared peptides were allocated to isoforms to obtain the final spectral counts, as (2) shows.

The MS-based protein abundance feature originates from spectral count, which, in the simplest form, counts the number of MS spectra assigned to one protein as its abundant feature. However, it is reportedly inaccurate in determining large fold change [16] or with low SCs [17, 18]. Therefore, other MS abundance features, such as MS/MS TIC, should be built for further analysis. freeQuant employs TIC as another MS abundance feature coupled with SC for quantification of complex proteomes to address the inherent deviation and systematic errors between replicate MS measurements. The advantages of this strategy lie in the dramatic extension of attainably quantitative ratios by the incorporation of ion counts and the protein size issue eliminated by the adoption of SC as divisor [19]. In order to obtain MS/MS TIC of each identified protein, all MS raw data are extracted to obtain MS/MS files using RawXtract [20]. As (3) shows, all intensities of each protein are cumulated, named the Spectral Index (SI). A two-step normalization is then taken to SI to eliminate the quantification bias from protein length and mass sampling. The normalized process, as a routine operation to eliminate systematic errors and inherent variances, can only be applied in some certain circumstances, for instance, when comparing relative changes between two complex mixture samples [19]. Thus, protein length was used to adjust protein size and all algorithms took two normalizing steps. Consider
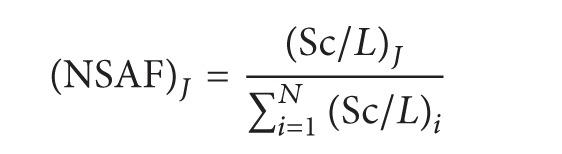
(1)

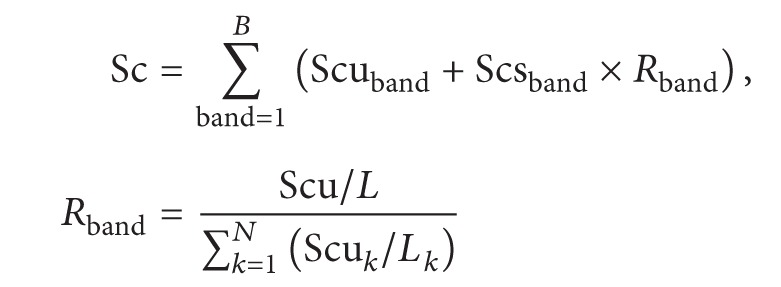
(2)

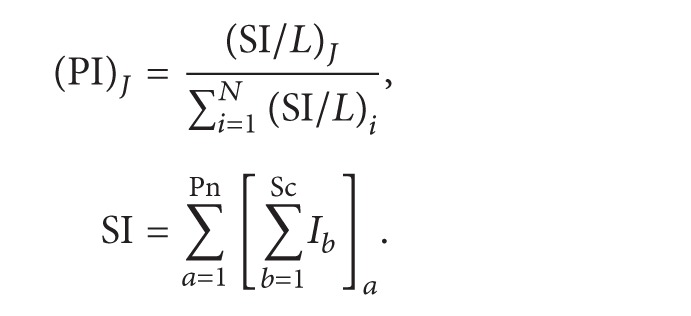
(3)Equation (1) is the definition of NSAF, where Sc is the spectral count of protein *J* and *L* is the length of protein *J* and *N* is the total number of proteins; (2) is the description of shared peptides, *B* is the total bands, Scu is the count of distinct peptides, Scs is the count of shared peptides, and *R* is the proportional factor; (3) is the description of TIC, PI is the final quantitative result, SI is the spectral counts with total ion counts, Pn is the distinct peptide, and *I* is the total ion counts.

### 2.3. Mining Biomedical Knowledge of Complex Proteome

**Figure 2:**
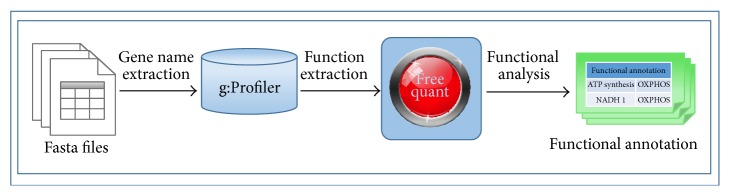
The functional analysis workflow. Gene name of each protein from IPI FASTA database was extracted by freeQuant. The list of gene name were inputted into g:Profiler via http://biit.cs.ut.ee/gprofiler/gconvert.cgi. A list of functional annotation results for each identified protein in Excel format were then generated by g:Profiler. Each functional annotation result was labeled with *p* value using Benjamini-Hochberg statistic method to show its statistical confidence. Next, the output of g:Profiler in Excel format was analyzed by freeQuant to automatically obtain functional annotation for complex proteomes. Especially in case of g:Profiler generating multiple functional annotation results for a single protein, the annotation result with smallest *p* value was finally selected to annotate the corresponding protein.

An important feature of freeQuant is to support functional analysis of complex proteomes. It is highly interactive to parse analytical results through g:Profiler. g:Profiler is a web-based bioinformatics tool for functional profiling of gene lists from large-scale experiments. It adopts the Benjamini-Hochberg statistic method to control False Discovery Rate (FDR) [21] and improve accuracy. According to these properties, g:Profiler is employed to obtain functional annotation of complex proteomes. Gene name of each protein was extracted from IPI FASTA database with our in-house toolkit and was analyzed through g:Profiler, then outcomes were analyzed by freeQuant to automatically annotate to obtain functional annotation of complex proteomes, and the workflow is shown in Figure 2. Importantly, *p* value was regarded as the key factor to filter functional annotations. For the protein with multiple functions, the functional annotation corresponding to the smallest *p* value was filtered. Therefore, large-scale functional annotations of complex proteomes were qualified and distributed by freeQuant.

## 3. Data Presentation, Visualization, and Analysis

**Figure 3:**
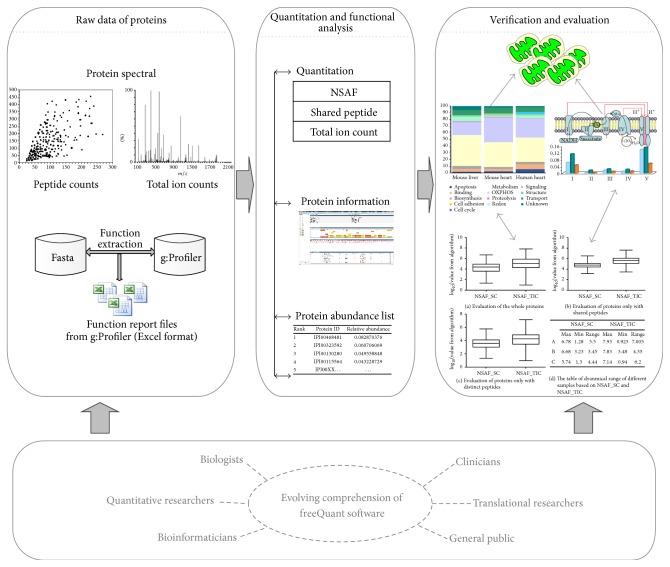
The data analysis pipeline with freeQuant. Three steps were involved. First, raw data were extracted to obtain spectral counts and total ion counts; functional profiling files were achieved through g:Profiler. Second, quantification coupled with biomedical knowledge was analyzed with freeQuant software toolkit using NSAF, MS/MS TIC, and shared peptides. Final, mitochondrial proteins were quantified and qualified by freeQuant. freeQuant software tool was appreciated by biologists, bioinformatics, and so forth.

**Figure 4:**
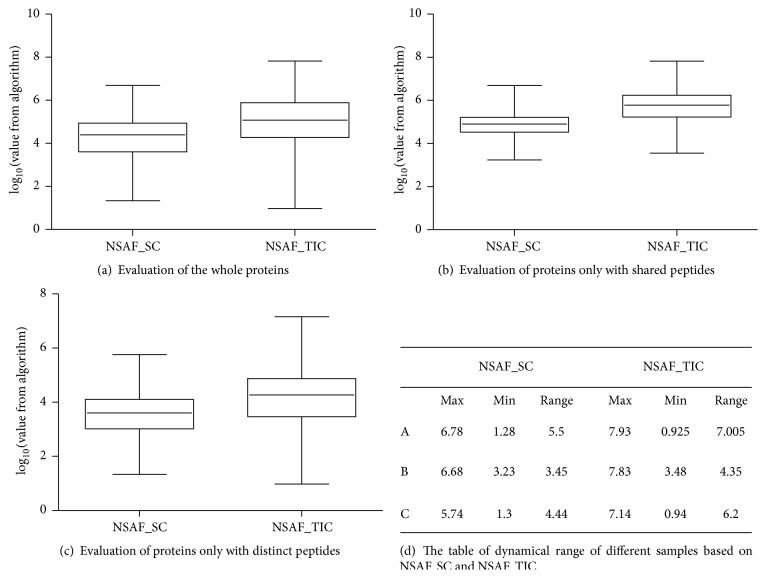
The protein abundance fold change of quantitative algorithm. Quantitative algorithms based on MS abundance features were involved. NSAF by spectral count and NSAF by total ion counts were used to quantify the whole protein dataset (a), proteins only with shared peptides (b), and proteins only with distinct peptides (c), respectively. It shows that the dynamical range of MS/MS TIC coupled with spectral count is broader than NSAF (d). Therefore, MS/MS TIC coupled with spectral count which alleviates this particular deficiency arising from SC was more suitable for determining protein abundance fold change. For panels (a), (b), and (c), the *x*-axis represents the two quantitative algorithms, NSAF by spectral count (NSAF_SC) and NSAF by TIC (NSAF_TIC); the *y*-axis represents the quantitative results of NSAF_SC or NSAF_TIC, which are calculated by Formula (1) and Formula (3) in Section 2.2, separately. The quantitative results are shown in log_10_ format.

With growing amounts of available MS-based data, the researcher's role in understanding, interpreting, and verifying quantitative and qualified results becomes ever more significant. Much effort has been put in developing quantitative accuracy of freeQuant and combining functional analysis of each identified protein for complex proteomes. In order to completely validate the accuracy and performance of freeQuant, mitochondrial proteins, which served as a classic type of complex proteomes, were selected for analysis. Mitochondria have received extensive attention due to their importance in cellular function including cell division, transport, metabolism, apoptosis, and known causative role in diseases. Mammalian mitochondria are double-membrane organelles, serving as the metabolic power houses of eukaryotic cells [14, 15, 22]. The data analysis pipeline is shown in Figure 3. Raw data from the mitochondrial MS experiments were searched using pFind protein search engine to obtain MS/MS spectra [23], and the MS/MS spectra were then extracted by RawXtract [20] to obtain total ion counts. For proteins with shared peptides, the count of shared peptides was used as an optimized factor to refine the abundance of mitochondrial protein isoforms. In parallel, functional annotation was automatically performed by freeQuant with the aid of g:Proflier. Based on the functional annotation results, the quantified mitochondrial proteins from the large-scale experiments were assigned to a total of 13 functional clusters which are highly related to the biological characteristics of mitochondria. To evaluate our proposed quantification method, the dynamic ranges of quantification results from both the total ion count and the spectral count were compared. It illustrated that the method with MS/MS TIC was more accurate than the method with SC only, as Figure 4 shows. Moreover, the new strategy with shared peptides was able to overcome the overestimated results, especially for the low-abundant protein isoforms, to improve the quantification accuracy, as Table 1 shows.

### 3.1. Assessment of Label-Free Quantitative Algorithm

#### 3.1.1. Data Acquisition and Preparation

All MS/MS spectra we used were from the previous work of the authors [24–26]; the workflow was shown as supplementary Figure S2. Mitochondria were treated with 0.5% DDM to extract membrane proteins and separated by SDS-PAGE followed by CBB G250 staining. Bands were sequentially cut from the continuum of the gel lane and were labelled to obtain much more accurate results in the peptide shared quantification. Proteins were digested with trypsin, and peptides were analyzed by LTQ-Orbitrap. Then, all MS/MS spectra including mouse heart mitochondrial dataset, mouse liver mitochondrial dataset, and human heart mitochondrial dataset were searched against the IPI mouse database (version 3.47) and IPI human database (version 3.68) [27] using pFind (version 2.6). Meanwhile, all MS raw data were searched through RawXtract (version 1.9.1) to obtain MS/MS files with fragment ion intensity. Detailed search parameters were performed the same as the authors' previous work [25]: partial tryptic digest allowing two missed cleavages, fixed modification of cysteine with carbamidomethylation (57.021 Da) and variable modification of methionine with oxidation (15.995 Da), and the precursor and fragment mass tolerances were set up at 1.5 and 0.5 Da, respectively. Peptides matching the following criteria were used for protein identification: DeltaCN ≥ 0.1; FDR ≤ 1.0%; peptide mass was 600.0~6000.0; peptide length was 6~60.

#### 3.1.2. Calculation of Protein Abundance Fold Change

Based on the data analysis pipeline, the applicability of label-free quantitative algorithms we proposed was investigated. Their abilities to calculate protein abundance fold change with regard to accuracy were tested with mitochondrial proteins. To get a clear view on the variability of these algorithms, we used the mass spectrometry data of mouse heart mitochondria to evaluate the dynamic range of each algorithm. Abundant features calculated were averaged and plotted in a boxplot to demonstrate the magnitude of each algorithm. The dynamic ranges of two starting abundance features were in the descending order as TIC and SC. Then, three groups of proteins based on different conditions including the whole proteins, proteins only with shared peptides, and proteins only with distinct peptides were analyzed by the two proposed algorithms, as shown in Figures 4(a), 4(b), and 4(c). We found that, as shown in Figure 4(d), the dynamical range of relative protein levels based on TIC values was broader than NSAF; particularly the estimation of MS/MS TIC was accurate over three orders of magnitude compared to NSAF. It was reported that if the dynamic range is much broader, more proteins will be quantified and the quantitative results are more accurate. Therefore, it demonstrated that TIC coupled with spectral count showed more accurate results than SC-based algorithm and it alleviated this particular deficiency arising from SC which was more suitable for determining protein abundance fold change. Meanwhile, TIC-based algorithm coupled with spectral count could avoid the inherent variance and systematic errors effectively between replicate MS measurements.

#### 3.1.3. Accuracy of Label-Free Quantitative Approach with Shared Peptides

SC, defined as the total number of spectra identified for a single protein, has recently gained acceptance. We evaluated the label-free approach based on SC, especially the new strategy with shared peptides. The MS data identified from the mouse heart mitochondria was used for the evaluation [24–26], and the MS experiments were repeated four times, generating four groups of MS/MS data, which were named as Group A, Group B, Group C, and Group D. All groups were searched by pFind and quantified by freeQuant to identify total counts of proteins. Based on the idea of NSAF, each group was quantified by the new strategy with shared peptides for further study, and all proteins were sorted in the descending order with regard to relative abundance. As supplementary Figure S3 shows, proteins with shared peptides accounted for 25%–40% of total proteins. We compared the quantification results between the NSAF method and our new method which introduced shared peptides for the protein families in the mitochondria, for example, the proteins from acyl-CoA dehydrogenase family. Proteins in this family play a significant role in life event due to their biochemical properties of fatty acid metabolism and lipid metabolism. As Table 1 shows, the proportion of proteins with shared peptides reached 90%. Ranking of these proteins in the family generally ascended after approaching by new strategy. Additionally, if all peptides of a protein were shared, the quantitative results were completely different, such as Acads. Consequently, we concluded that normalized processes we designed eliminated systematic errors and should be considered when dealing with MS/MS spectra. Simultaneously, the new strategy with shared peptide overcame inaccurate and overestimated results for low-abundant isoforms.

### 3.2. Biomedical Knowledge Annotation against Quantitative Proteomics

#### 3.2.1. Functional Clustering and Distribution of Identified Proteins

**Figure 5:**
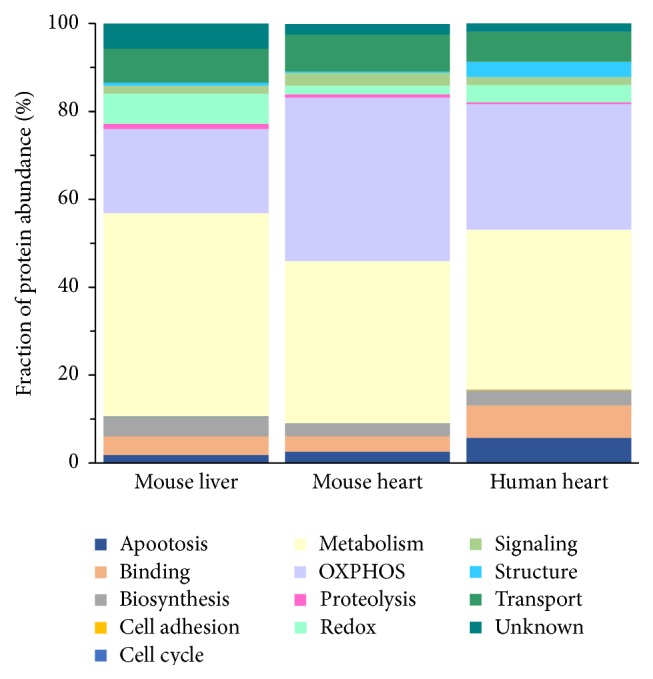
The analysis of functional clustering for mitochondrial proteins. Three mitochondrial proteins were involved including mouse heart mitochondrial dataset, mouse liver mitochondrial dataset, and human heart mitochondrial dataset. *x*-axis represents different samples, *y*-axis represents fraction of protein abundance, and the different colours represent different functional clusters. Functional annotations were distributed to 13 clusters due to biomedical properties of mitochondria. Metabolic proteins show highest abundance in mouse liver mitochondrial dataset, while oxidative phosphorylation proteins show highest abundance in cardiac mitochondrial dataset.

Next, we sought to understand the protein functions of the complex proteome using g:Profiler, which is able to annotate the protein functions from the Gene Ontology Annotation (GOA) database [28], and all the annotation results output from g:Profiler were automatically parsed with freeQuant. In order to make the functional cluster closer to the biochemical properties of mitochondrial proteins, we assigned all identified mitochondrial proteins to a total of 13 functional clusters according to the summary of our previous study [25]. These 13 functional clusters include apoptosis, DNA/RNA/protein synthesis, metabolism, oxidative phosphorylation, protein binding/folding, proteolysis, redox, signal transduction, structure, transport, cell adhesion, and cell cycle. The proteins which were unable to assign functional group were therefore classified as “unknown,” as Figure 5 shows. Metabolic proteins have highest abundance in mouse liver mitochondrial dataset, while oxidative phosphorylation proteins show highest abundance in cardiac mitochondrial dataset. This explains that liver plays a vital role in metabolic process including nutrients synthesis, transformation, and decomposition. However, heart promotes blood flowing to provide adequate blood to the organs or tissues, supplies oxygen or various nutrients, and takes metabolic products away. Functional clustering for complex proteomes contributes to comprehending physiological and pathological characteristics of mitochondrial proteins.

We, respectively, selected top 10 most abundant proteins in each sample, from this point of functional analysis, and these proteins play a major role in Electron Transport Chain (ETC) complex, metabolism, tricarboxylic acid cycle, and so forth. Top 10 most abundant proteins in human heart mitochondrial dataset which show significant function were listed, as supplementary Table S1 shows. Similarly, it was found that those proteins show high abundance in mouse heart mitochondria and six of them also rank top 10 especially. However, two of them which are from ETC complex rank top 10 in mouse liver mitochondria, and the relative abundance of the rest of them is extremely low. Then top 10 proteins in mouse heart mitochondria were analyzed, as supplementary Table S2 shows, and four of those proteins show high relative abundance in human heat mitochondrial dataset. For proteins in mouse liver mitochondrial dataset, as supplementary Table S3 shows, the distribution of such proteins shows great difference; some individual proteins even cannot be identified, as IPI00420718. Furthermore, the protein which ranks the first (IPI00111908) in mouse liver mitochondria ranks 999 in mouse heart mitochondria and ranks 1004 in human heat mitochondria, respectively. It lies in the protein which almost participates in urea cycle merely existing in liver in mammals. By this token, functional distribution based on quantification contributes to detecting differential protein expression and quantitative proteomics is not only a way for data processing but also an important approach for exploring protein functions and interactions in a large-scale manner for complex proteomes.

#### 3.2.2. Quantitative Analysis of Mitochondrial ETC Subunits

**Figure 6:**
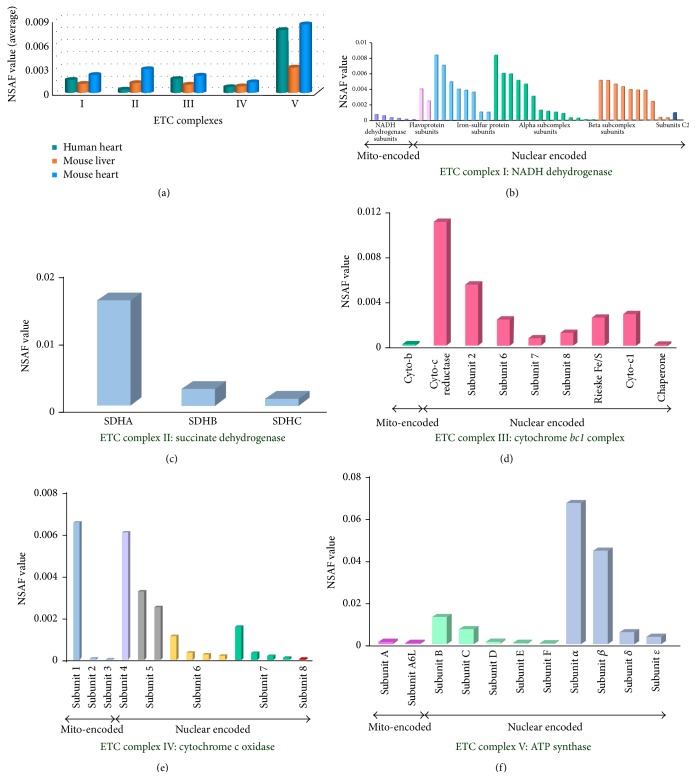
The quantitative analysis of ETC complex subunits. Three groups of proteins with different complexes were compared with averaged NSAF of each ETC complex. (a) shows quantitative abundance of all five ETC complexes; Complex I to Complex IV displayed very similar level of abundances. In comparison, Complex V appeared to be in higher abundance, approximately 3-fold more than others. (b) shows quantitative analysis of proteins in Complex I and proteins were encoded by mitochondrial genome or nuclear genome, respectively; (c) shows quantitative analysis of proteins in Complex II; (d) shows quantitative analysis of proteins in Complex III and proteins were encoded by mitochondrial genome or nuclear genome, respectively; (e) shows quantitative analysis of proteins in Complex IV and proteins were encoded by mitochondrial genome or nuclear genome, respectively; (f) shows quantitative analysis of proteins in Complex V and proteins were encoded by mitochondrial genome or nuclear genome, respectively.

In the mitochondria of eukaryotic cells, ETC subunits consist of a series of redox reactions in which electrons are transferred from a donor molecule to an acceptor molecule. The change of abundance in ETC subunits may cause mitochondrial dysfunction. Our study shows that ETC complexes are highly abundant in different biological species and tissues. A much detailed analysis can draw a complete picture regarding the distribution of ETC subunits abundance. We classified proteins among ETC complexes and then compared averaged NSAF of each ETC complex. As Figure 6(a) shows, the relative abundance of Complex V which is the main factory for synthesizing ATP shows higher abundance than others. Our analysis illustrated that, among all five ETC complexes, Complex I to Complex IV mainly participate in the process of mitochondrial inner or outer membrane electron transport, while Complex V is used for synthesizing ATP. Meanwhile, Complex I to Complex IV display very similar level of abundances. In comparison, Complex V appears to be in higher abundance, which is approximately 3 times more than others. Then we selected mouse heart mitochondria for further study (Figures 6(b), 6(c), 6(d), 6(e), and 6(f)). Not surprisingly, the majority of proteins identified in our study are encoded by the nuclear genome, while 12 of the ETC subunits are encoded by mitochondrial genome [25, 29]. They are MTATP6, MTATP8, MTCO1, MTCO2, MTCO3, MTCYB, MTND1, MTND2, MTND3, MTND4, MTND4L, and MTND5, respectively [30]. These 12 ETC subunits show significantly low abundance when compared to the nuclear-encoded ETC subunits, suggesting that these mitochondrial-encoded proteins are the limited factors for effective assembly of the ETC complex. More importantly, some related research reports indicate that many diseases caused by lacking of mitochondrial function are highly correlated with these proteins encoded by mitochondrial genome [31].

#### 3.2.3. Quantitative Analysis of Mitochondrial Interactome

**Figure 7:**
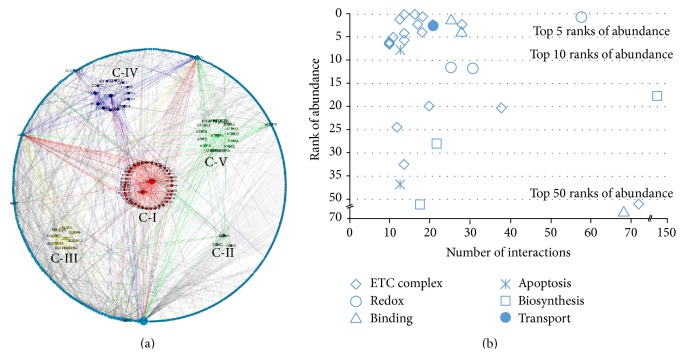
(a) The quantitative analysis of mitochondrial interactome. Each node represents a protein, the size of each mitochondrial node represents the number of connections in the protein-protein interaction network, and the larger nodes represent more interactions. (b) The analysis of top 30 proteins in mitochondrial interactome. Different types represent different functions, *x*-axis represents number of interaction, *y*-axis represents rank of abundance, 17 of them are from ETC complex, and almost all proteins are top 50 high abundance and, especially, 18 proteins are top 10 high abundance.

Human heart mitochondrial proteins were selected and queried within IntAct database for identified protein-protein interactions to demonstrate quantification coupled with functional analysis for complex proteomes [32]. Cytoscape 3.0 Network Data Integration, Analysis, and Visualization software was then utilized to display interactions among all proteins [33].  Figure 7(a) shows the mitochondrial interactome network combined with quantitative analysis results. Each node represents a protein, the size of each mitochondrial node represents the number of connections in the protein-protein interaction network, and the larger nodes represent more interactions. In addition, we listed the top 30 proteins of mitochondrial interactome in Figure 7(b); 17 of these proteins are ETC complex subunits. This study shows that Complex I proteins were centralized around NADH-ubiquinone oxidoreductase AB1 subunit (NDUFAB1), a feature highlighting the importance of NDUFAB1 as a core protein; another kernel protein in Complex I subunits was NADH-ubiquinone oxidoreductase B8 subunit (NDUFA2), and the rest of the proteins in Complex I had a total of 57 interactions. In contract, the Complex II subunits showed small scattered clusters and fragmented groupings. The Complex III proteins were centralized around CYC1, its important role in OXPHOS. The Complex IV proteins showed similar interactions, and the core protein of Complex IV was COX4I1. In the Complex V interactome, ATP5F1 and ATP5B demonstrated the most protein-protein interactions and ATP5B served as the major hub in the human heart mitochondrial proteins. Beside ETC subunits, other high-abundant mitochondrial proteins also show numerous protein-protein interactions, such as ICT1, IMMT, ALDCA, SCL25A13, ACADVL, and ACAD9. ICT1 is an essential protein necessary for mitochondrial protein synthesis. A total of 143 protein-protein interactions in our study were detected, ranking as top 1 of the whole mitochondrial proteome, illustrating that ICT1 serves as the major hub in mitochondrial interactome. Richter et al. (2010) show that mammalian mitochondrial ribosomes contain a ribosomal protein (ICT1) that acts as a ribosome-dependent, codon-independent peptidyl-tRNA hydrolase. This ribosomal protein can rescue ribosomes stalled on mRNAs lacking a termination codon [34, 35]. Another vital protein was ACADVL, which shows 63 protein-protein interactions. Its important role is redox which exists in mitochondria only. Mutations in the ACADVL gene lead to a shortage of VLCAD enzyme within cells. Without sufficient amounts of this enzyme, very long-chain fatty acids are not metabolized properly. Inborn error of long-chain fatty acid metabolism is often characterized by cardiac hypertrophy, arrhythmia, and sudden death in the disease phenotype of affected children [36, 37]. Meanwhile, it was reported that these results were indicative of the intergenomic protein-protein properties, which may have been influenced by the functional analysis [38]. Subsequently, mitochondrial interactome coupled with quantification and functional analysis were analyzed. As Supplementary Table S4 shows, in the aspect of functional analysis, half of these proteins were from ETC complex and participated in synthesizing energy ATP, and the rest also played a vital role in redox, binding, transport, OXPHOS, signaling, and biosynthesis; protein-protein interaction was entwined with protein abundance, almost all proteins are top 50 and, especially, 18 proteins are top 10. The integrated framework addressing protein interactions with quantification may elucidate mechanistic insights that play a fundamental role in targeting disease and contributes to providing a new way to discover other crucial proteins.

## 4. Conclusions

freeQuant, which addresses the abovementioned challenges in complex proteomes, consists of two well-integrated modules: the kernel part is label-free quantitative algorithm supporting kinds of label-free quantitative approaches, such as NSAF-based on spectral count, NSAF with shared peptides, and TIC coupled with spectral count; another important feature is the combination quantification with functional analysis, which is a meaningful way to deeply explore interactions and relationships for complex proteomes. We have already applied freeQuant to quantify and qualify mitochondrial proteins and analyzed quantitative results based on biomedical knowledge. The results demonstrated that MS/MS TIC coupled with spectral count can lower inherent variances between replicate MS experiments and has broader dynamical range to improve accuracy, especially it is capable of alleviating this particular deficiency arising from SC which is more suitable for determining protein abundance fold change, and the new strategy with shared peptides overcomes inaccurate and overestimated results for low-abundant isoform. Label-free quantitative approaches coupled with functional analysis can thoroughly comprehend the relationship and correlation of complex proteomes in large-scale experiments and contribute to providing a new method for complex proteomes analysis.

freeQuant has additional features. It adopts wizard dialog to help users load data and it uses parallel computing to shorten time to quickly process large-scale data. Different visualization methods were employed to display analytical results. The home page describes all the information of a protein, such as basic protein information, spectral abundance atlas, sequence, and distinct peptides, and the comparative page profiles the rank list of whole proteins with regard to relative abundance to reveal the differences of proteins abundance as a whole. In conclusion, freeQuant provides ease-to-use interface and accurate quantification method for the proteomics science community to study the complex proteome.

## Supplementary Material

Tables S1, S2, and S3 list the top 10 most abundant proteins in the human heart mitochondrial dataset, the mouse heart mitochondrial dataset, and the mouse liver mitochondrial dataset respectively. Table S4 lists the top 30 most interactions proteins in the mitochondrial interactome.
Figure S1 shows the functional modules of freeQuant. Figure S2 shows the workflow of mitochondrial data acquisition and preparation in this study. Figure S3 shows the percentage of proteins with shared peptides in four groups of mouse heart mitochondrial proteome dataset.

## Figures and Tables

**Table 1:** The analysis of proteins in acyl-CoA dehydrogenase family.

Protein name	Total count of peptides	Total count of shared peptides	Rate (%)	Rank (NSAF only)	Rank (with shared peptides)
Acadvl	3033	3000	98.91	8	8
Acadl	1449	1419	97.93	11	12
Acadm	1150	1131	98.35	51	39
Acads	705	692	98.16	63	63
Acad8	200	188	94.00	140	100
Acads	180	180	100	157	1444
Acad9	155	144	92.90	182	111
Acadsb	113	105	92.92	228	165
Acad10	85	80	94.12	395	289

## Abbreviations

freeQuant: Quantitative proteomics comparison
MS: Mass spectrometry
NSAF: Normalized spectral abundance factor
SC: Spectral count
IPI: International protein index
FDR: False discovery rate
TIC: Total ion count
ETC: Electron transport chain.
