# Supplementary material for: freeQuant: A Mass Spectrometry Label-Free Quantification Software Tool for Complex Proteome Analysis
Source: ScientificWorldJournal. 2015 Nov 8;2015:137076. doi: 10.1155/2015/137076 (PMC4667154; doi:10.1155/2015/137076)
Supplement: Supplementary file 1 — Tables S1, S2, and S3 list the top 10 most abundant proteins in the human heart mitochondrial dataset, the mouse heart mitochondrial dataset, and the mouse liver mitochondrial dataset respectively. Table S4 lists the top 30 most interactions proteins in the mitochondrial interactome. Figure S1 shows the functional modules of freeQuant. Figure S2 shows the workflow of mitochondrial data acquisition and preparation in this study. Figure S3 shows the percentage of proteins with shared peptides in four groups of mouse heart mitochondrial proteome dataset. [file 137076.f1.pdf]

freeQuant: a mass spectrometry label-free quantification software tool for complex proteome analysis

Supplement

Ning Deng[1 §], Zhenye Li[2], Chao Pan[1], Huilong Duan[1]

[1] Department of Biomedical Engineering, Key Laboratory for Biomedical Engineering of Ministry of Education, Zhejiang University, Hangzhou 310027, China
[2] General Hospital of Ningxia Medical University, Yinchuan 750004, China

[§] **Correspondence:**
Ning Deng
Department of Biomedical Engineering
Key Laboratory for Biomedical Engineering of Ministry of Education,
Zhejiang University, Hangzhou, 310027, China.

Supplementary Tables

Table S1-The Top 10 Most Abundant Proteins in Human Heart Mitochondrial Dataset

Rank	IPI	Gene	Function	Rank Mouse Heart	Rank Mouse Liver
1	IPI00303476	ATP5B	ETC complex	1	3
2	IPI00440493	ATP5A1	ETC complex	2	4
3	IPI00022314	SOD2	Redox	32	27
4	IPI00017855	ACO2	Metabolism	3	55
5	IPI00015141	CKMT2	Transport	14	14
6	IPI00022891	SLC25A4	Transport	5	59
7	IPI00007188	SLC25A5	Transport, Apoptosis	121	36
8	IPI00015911	DLD	TCA cycle	222	631
9	IPI00337541	NNT	Metabolism	7	202
10	IPI00291006	MDH2	TCA cycle,Metabolism	4	17

Table S2-The Top 10 Most Abundant Proteins in Mouse Heart Mitochondrial Dataset

Rank	IPI	Gene	Function	Rank Human Heart	Rank Mouse Liver
1	IPI00468481	Atp5b	ETC complex	1	3
2	IPI00130280	Atp5a1	ETC complex	2	4
3	IPI00116074	Aco2	TCA cycle	5	45
4	IPI00323592	Mdh2	Metabolism	14	14
5	IPI00115564	Slc25a4	Transport	7	48
6	IPI00223092	Hadha	Fatty Acid Metabolism	15	16
7	IPI00309964	Nnt	OXPHOS	13	163
8	IPI00119203	Acadvl	Metabolism	25	23
9	IPI00230351	Sdha	OXPHOS	180	41
10	IPI00226430	Acaa2	Metabolism	73	2

Table S3-The Top 10 Most Abundant Proteins in Mouse Liver Mitochondrial Dataset

Rank	IPI	Gene	Function	Rank Mouse Heart	Rank Human Heart
1	IPI00111908	Cps1	Urea cycle	999	1004
2	IPI00226430	Acaa2	Fatty Acid Metabolism	10	65
3	IPI00468481	Atp5b	ETC complex	1	1
4	IPI00130280	Atp5a1	ETC complex	2	2
5	IPI00420718	Hmgcs2	TCA cycle	N/D	N/D
6	IPI00116753	Etfa	ETC complex	27	36
7	IPI00308885	Hspd1	Binding	15	23
8	IPI00111218	Aldh2	Redox	200	91
9	IPI00114710	Pcx	Binding	114	824
10	IPI00230507	Atp5h	ETC complex	12	13

Table S4- The top 30 most interactions proteins in mitochondrial interactome

Protein Name	Degree of Interaction	Relative Abundance	Rank of Abundance (%)	Function
ICT1	143	0.00039753	16.3	Biosynthesis
NDUFAB1	79	0.000017185	53.87	ETC Complex
ALDOA	69	0.000007367	69.67	Binding
ACADVL	63	0.016066891	0.566	Redox
NDUFA2	41	0.000257252	20.2	ETC Complex
C1qbp	33	0.000779924	11.64	Binding
COX4I1	30	0.006068686	2.20	ETC Complex
IMMT	30	0.004301678	3.65	Binding
ACAD9	27	0.000792398	11.45	Redox
HSPD1	27	0.011790555	0.97	Binding
MRPL10	23	0.000121663	27.88	Biosynthesis
ATP5F1	22	0.006868338	1.96	ETC Complex
SLC25A13	22	0.005681322	2.52	Transport
COX6C	21	0.000264556	19.76	ETC Complex
SDHA	19	0.01555497	0.566	ETC Complex
NDUFS3	19	0.003925022	3.84	ETC Complex
MT-CO2	18	0.006538737	2.08	ETC Complex
ATP5B	17	0.066614011	0.063	ETC Complex
MRPL16	17	0.000017704	53.37	Biosynthesis
ATP5A1	14	0.044037116	0.126	ETC Complex
NDUFS2	14	0.003784353	4.09	ETC Complex
NDUFAF1	14	0.000081823	32.47	ETC Complex
CYC1	14	0.002803127	5.48	ETC Complex
VDAC1	13	0.000061074	36.69	Apoptosis
UQCRC1	13	0.011025876	1.01	ETC Complex
SLC25A5	13	0.0015723	7.61	Apoptosis
COX6A2	12	0.000165825	24.42	ETC Complex
COX5A	11	0.00326276	4.91	ETC Complex
UQCRFS1	10	0.002505937	5.92	ETC Complex
NDUFV2	10	0.00240998	6.36	ETC Complex

Supplementary Figures

Figure S1– The Main Framework of freeQuant

freeQuant consists of four parts: data loading, quantification, functional analysis and results displaying.

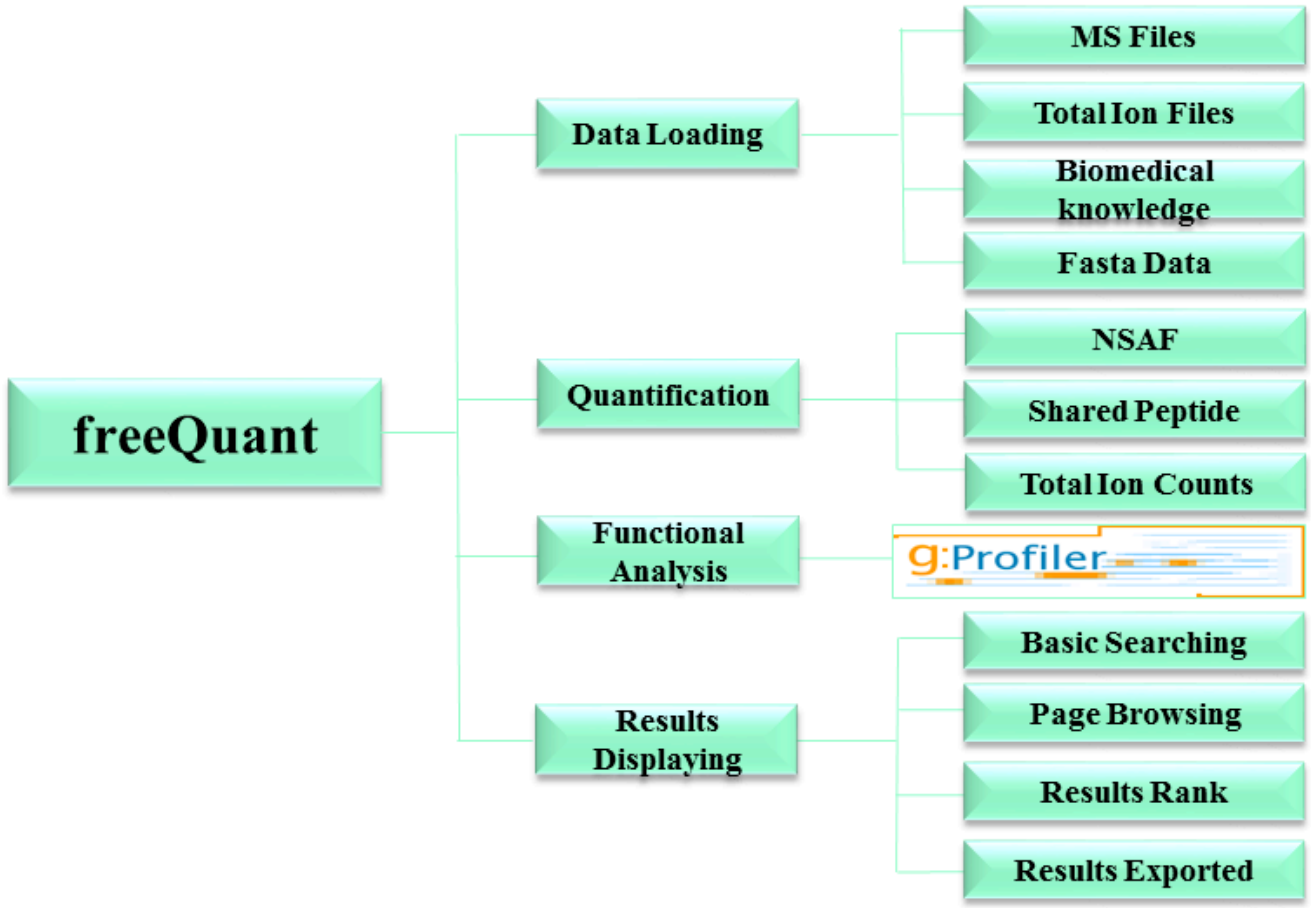

Figure S2– The Workflow of Data Acquisition and Preparation

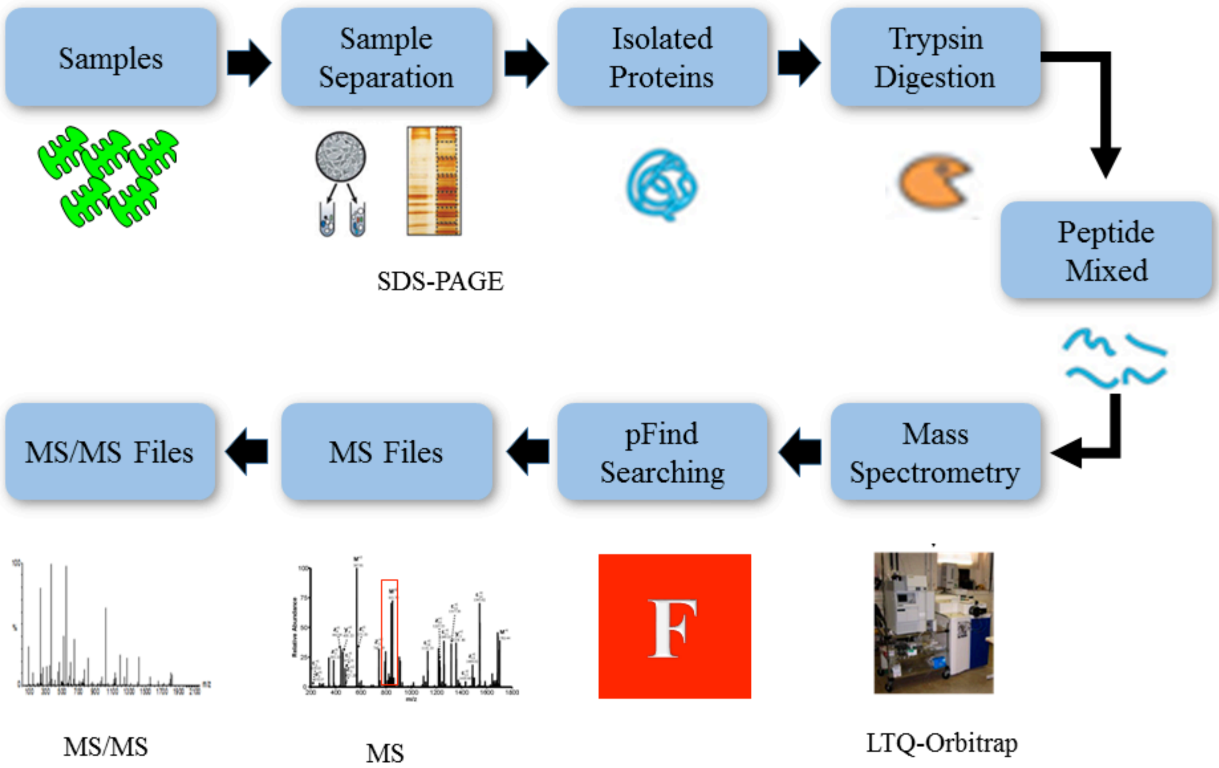

Figure S3– The Analysis of Proteins with Shared Peptides in 4 Groups

Four groups of mouse heart mitochondrial proteins were quantified with NSAF and NSAF_S. Proteins with shared peptide were accounted twenty five to forty percent of total protein.

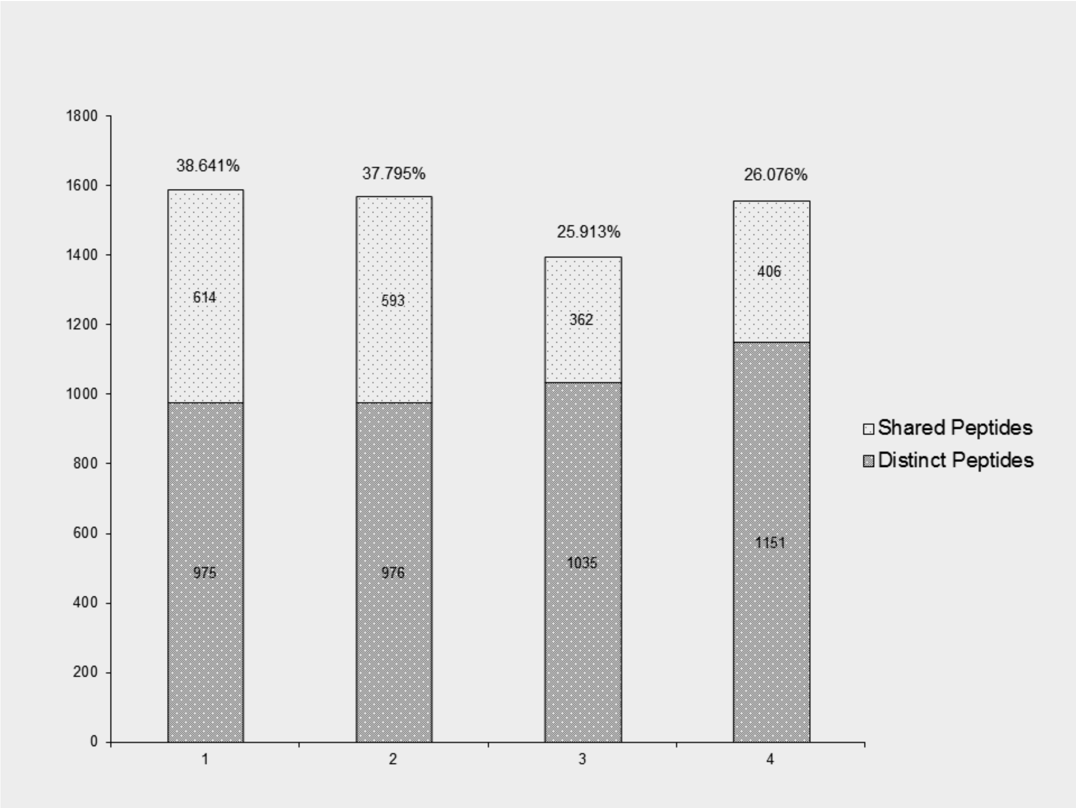

Supplementary Notes

The freeQuant software tool is free available via ftp://ftp.vico-lab.com/.
